# Complete transperitoneal laparoscopic nephroureterectomy in a single position for upper urinary tract urothelial carcinoma and comparative outcomes

**DOI:** 10.1186/s12957-021-02297-0

**Published:** 2021-07-02

**Authors:** Chengwu Xiao, Yang Wang, Meimian Hua, Wei Zhang, Guanyu Ren, Bin Yang, Qing Yang

**Affiliations:** Department of Urology, Changhai Hospital, Navy Medical University, 168 Changhai Rd, Shanghai, 200433 China

**Keywords:** Urinary tract urothelial carcinoma, Laparoscopy, Nephroureterectomy, Single position

## Abstract

**Background:**

To describe the techniques and outcomes of complete transperitoneal laparoscopic nephroureterectomy (CTLNU) for upper urinary tract urothelial carcinoma (UTUC) in a single position.

**Materials and methods:**

Those patients with localized UTUC were included, among which 50 cases had CTLNU while 48 cases had laparoscopic nephroureterectomy with open bladder cuff excision (LNOBE). The clinical data were collected and analyzed retrospectively.

**Results:**

All 98 patients underwent successful procedures of radical nephroureterectomy without transferring into open surgery. No significant difference was found among baseline clinical characteristics. Compared with the LNOBE group, the CTLNU group had a shorter operative time (98.5±40.3 min vs. 132.4±60.2 min), less blood loss (60.4±20.3 ml vs. 150.6±50.2 ml), shorter length of hospital stay (5.3±2.2 days vs. 8.1±2.3 days), and shorter incision (6.3±1.2 cm vs. 11.5±3.2 cm). The disease-related outcomes such as pathological stage, tumor grade, and recurrence rate were similar between the two groups.

**Conclusions:**

The CTLNU in a single position had advantages of shorter operation time, less blood loss, and shorter incision length. This surgical technique is a more minimally invasive, simplified, and effective way to perform the radical nephroureterectomy.

## Background

Upper urinary tract urothelial carcinoma (UTUC) accounts for only 5–7% of all urothelial carcinoma cases [1]. The tumor is often discovered in the renal pelvis with bladder recurrences being frequently diagnosed. Radical nephroureterectomy combined with bladder cuff excision is the “gold standard” surgery procedure for upper UTUC [2]. Traditional open nephroureterectomy (ONU) with excision of bladder cuff around the ureteral orifice required two surgical incisions, which is associated with significant postoperative pain [3, 4].

As one of the surgical patterns for UTUC, laparoscopic nephroureterectomy with open bladder cuff excision (LNOBE) has its drawbacks in that two positions cannot be avoided during the surgery and a large midline incision in the lower abdomen for bladder cuff resection is required. The advantage of complete transperitoneal laparoscopic nephroureterectomy (CTLNU) is that it does not require repositioning and resterilizing, making it a minimally invasive, safe, and effective surgery procedure [5]. The aim of our study is to introduce this novel technique, which can reduce surgical trauma and promote faster recovery of patients.

## Methods

### Patients

Between January 2016 and June 2019, 98 patients whose UTUC being diagnosed by pathology or imaging were included in our study. All patients were preoperatively evaluated using imaging examinations including IVU, CT or MRI, and cystoscopy. The urothelial carcinoma cells were discovered by urine cytology in 91 patients. In addition, 7 cases whose imaging and cytology diagnosis was uncertain underwent ureteroscopy with biopsy. The baseline characteristics, demographic data, tumor stage, and histological grade were recorded and compared between CTLNU and LNOBE groups.

### Operating technique

#### CTLNU

**Fig. 1:**
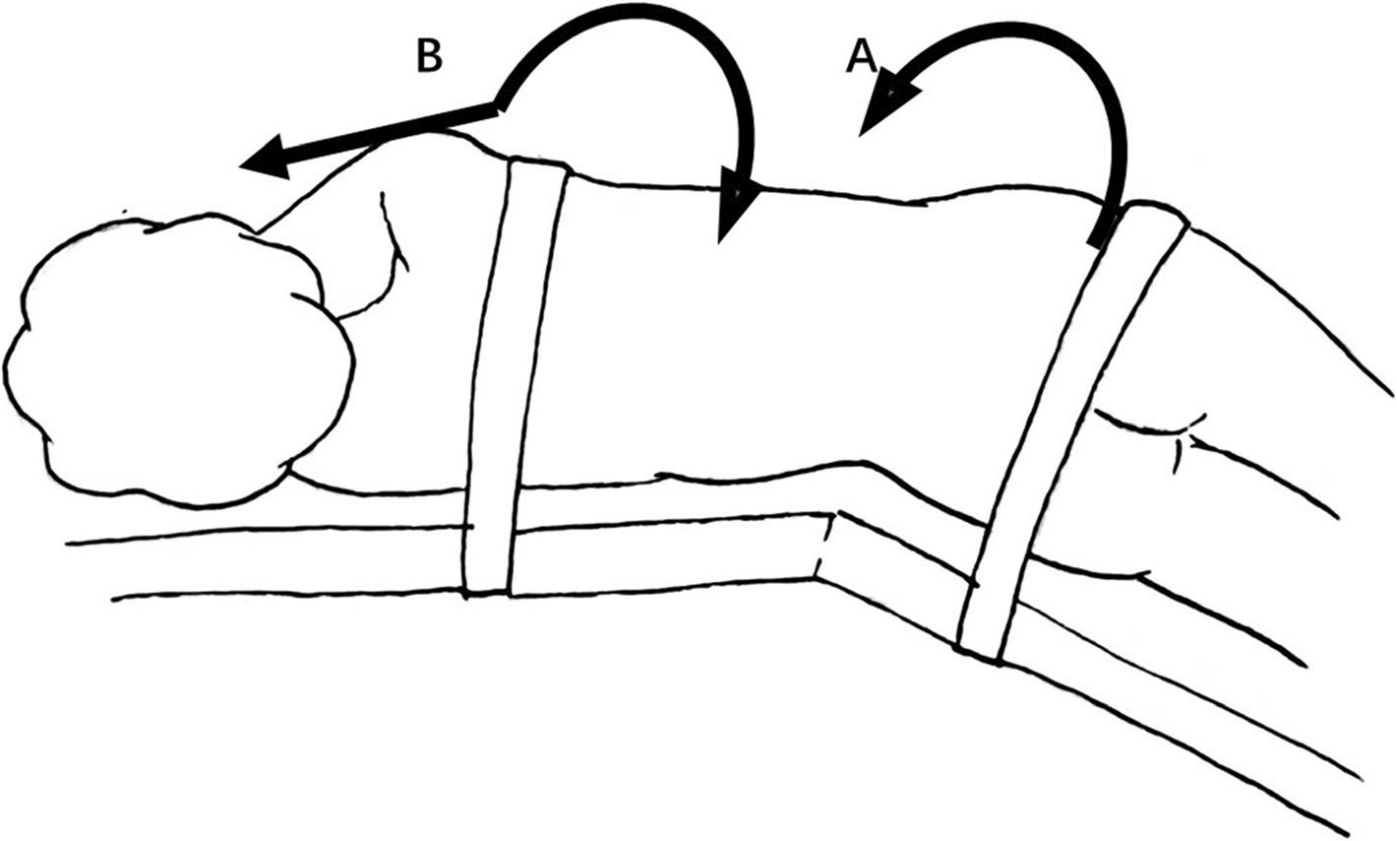
The position for operation (the patient was placed in a 45-degree flank position to confirm safe; (**A**) rotate operation bed 30° to the ventral side for kidney and proximal ureter dissection; (**B**) rotate operating bed 30° to the dorsal side, head low, and foot high for distal ureter and bladder cuff dissection)

**Fig. 2:**
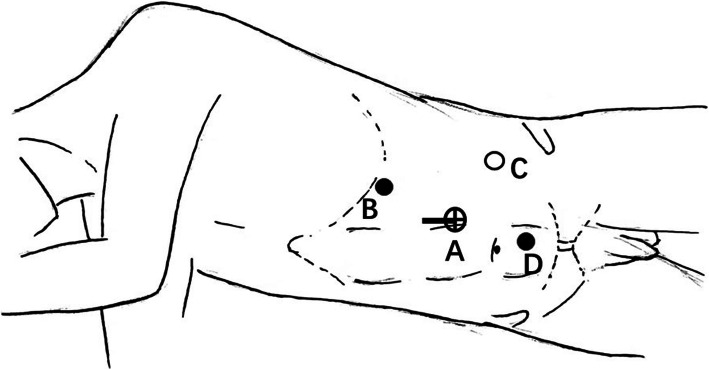
Trocar arrangement and incision for specimen extraction (left side) for CTLNU: (**A**) camera port: a 10-mm trocar is placed lateral margin of the rectus abdominis muscle of the first finger above the umbilicus; (**B**) a 5-mm trocar is placed costal margin along the mid-clavicular line; (**C**) a 12-mm trocar is placed midpoint of the line between the anterior superior iliac spine and the navel; and (**D**) 5-mm trocar is inserted in the midline between the umbilicus and the xiphoid process. Specimen extraction is through an enlarged transrectus abdominal incision

After induction of general anesthesia, a nasogastric tube and transurethral catheter were respectively placed to decompress the stomach and bladder. The patient was placed in a 45-degree flank position to ensure their safety during the surgery (Fig. 1). Trocar arrangement and corresponding incision locations were similar to the standard radical transperitoneal laparoscopic nephrectomy (Fig. 2) [6, 7]. However, the camera port was located at the lateral margin of the rectus abdominis muscle among our cases. Further dissection into the pelvis can be achieved by insertion of an additional 5-mm trocar in the lower abdomen, which is located at the midline between the umbilicus and the pubic symphysis.

Before the start of surgery, the operating table was rotated 30° to the ventral side, a similar position for laparoscopic radical nephrectomy, which made the exposure of renal hilum and resection of the kidney much easier. Exposure of the surgical field is initially performed by incising along the Toldt’s line down to the pelvic brim and then mobilizing the bowel. In order to prevent the tumor distally seeding through the mobilization of the ureter towards the bladder, the ureter was identified, dissected, and clipped with hemo-lok at the pelvic brim. Following the dissection and cut off of the renal artery and vein, the kidney was completely excised meanwhile the perirenal fascia and adrenal gland were preserved.

After this, the operating table was further rotated 60° to the dorsal side, simultaneously keeping the head low and foot high, a similar position for the cystectomy to expose the lower ureter and bladder cuff. The direction of the camera and working trocars were changed towards the pelvis without the repositioning of patients. Ureter dissection was continued caudally till the detrusor muscle fibers were identified at the ureterovesical junction. The 1-cm-diameter bladder adventitia around the ureterovesical junction was cleared, and the bladder was incised longitudinally where the ipsilateral ureter was inserted. An adequate bladder cuff was then excised and the ipsilateral orifice was visually confirmed. The bladder defect was closed with continuous suture using a 3-0, 15-cm V-lock suture, secured at both ends with hemo-lok. Finally, the bladder was filled to check the water tightness.

The entire specimen was put into an impermeable organ bag and extracted en bloc through an enlarged transrectus abdominal incision. Two suction drains were respectively placed in the perivesical space and the perirenal space.

#### LNOBE

**Fig. 3:**
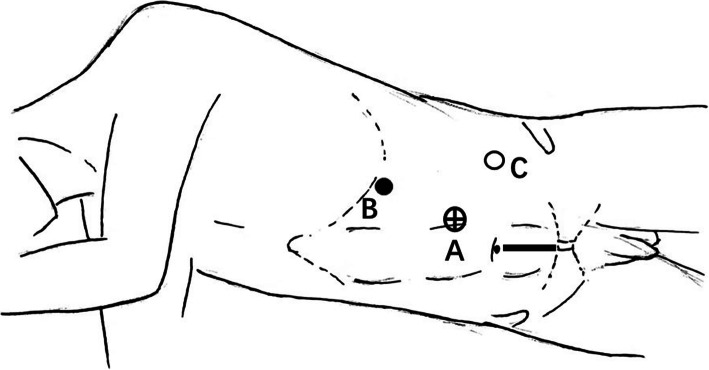
Trocar arrangement and incision for specimen extraction (left side) for LNOBE (**A**) camera port: a 10-mm trocar is placed lateral margin of the rectus abdominis muscle of the first finger above the umbilicus; (**B**) a 5-mm trocar is placed costal margin along the mid-clavicular line; (**C**) a 12-mm trocar is placed midpoint of the line between the anterior superior iliac spine and the navel. Specimen extraction is through the median incision of the lower abdomen

After the induction of general anesthesia, a nasogastric tube and transurethral catheter were respectively placed to decompress the stomach and bladder. The patients were placed in a 90-degree full flank position, and 3 laparoscopic ports were placed referring to the trocar arrangement of standard radical nephrectomy (Fig. 3). The ureter was also lipped with hemo-lok at the pelvic brim to prevent tumor seeding into the bladder. When the kidney was completely excised with the adrenal gland preserved, the patients were switched into a flat position, then redisinfected, and relayed the sheets.

After the median incision of the lower abdomen was made, the ureter was continuously dissected until the detrusor muscle fibers at the ureterovesical junction were identified. The 1-cm-diameter bladder adventitia around the ureterovesical junction was cleared and clamped with right-angled forceps, and then the ureteric orifice and bladder mucosa were resected. The defect of the bladder wall was closed with a continuous suture of muscle and mucosa using 3-0 absorbable suture. The entire specimen was extracted en bloc through a lower abdominal incision. Two suction drains were respectively placed in the perivesical space and the perirenal space.

### Postoperative care

All patients underwent immediate bladder irrigation with hydroxycamptothecin (15 mg dissolved in 50 mL physiological saline) in the initial 24 h after surgery. Follow-up time was calculated from the date of surgery to that of the most recent documented examination. A physical examination and cystoscopy were performed every 3 months in the first and second years, and every 6 months thereafter. Imaging examinations such as CT or MRI were performed every 6 months in the first year, and once a year thereafter.

### Statistical analysis

Propensity score matching was performed between two groups in these patients, and all the data was analyzed using the SPSS 23.0 software. The chi-square test or Fisher exact test was used to determine any significant differences in the normal data. The Student’s t-test was used to analyze differences in the continuous variables. Statistical significance was accepted if P value < 0.05.

## Results

All the laparoscopic procedures were successfully performed, without conversion to open surgery or any major complications. The baseline characteristics of patients are shown in Table 1. There was no significant difference in age, side, ASA score, gender, tumor location, and postoperative pain score among the patients who underwent CTLNU or LNOBE.

**Table 1:** Baseline characteristics and surgical outcomes of patients

	CTLNU	LNOBE	P value
No. of patients (n)	50	48	
Age (*x̅* ±s, years)	67±10.5	66±11.8	0.93
Side (R/L, n)	26/24	20/28	0.86
Gender (F/M, n)	30/20	25/23	0.92
Median ASA score (1/2/3, n)	2/47/1	3/45/0	0.91
Location of tumor (n)			0.89
Renal pelvis	43	38	
Ureter	7	10	
Operative time (*x̅* ±s, min)	98.5±40.3	132.4±60.2	<0.01
Blood loss (*x̅* ±s, ml)	60.4±20.3	150.6±50.2	<0.01
Postoperative pain score ( *x̅* ±s)			
24 h after surgery	1.93±1.2	2.04±1.5	0.22
48 h after surgery	1.17±1.1	1.25±1.6	0.32
Length of hospital stay (*x̅* ±s, d)	5.3±2.2	8.1±2.3	<0.01
Recovery of bowel function (*x̅* ±s, d)	1.8±0.8	2.1±0.7	0.89
Incision length (*x̅* ±s, cm)	6.3±1.2	11.5±3.2	<0.01
Pathological stage (T1/T2/T3, n)	6/23/1	5/18/1	0.86
Tumor grade (G1/G2/G3, n)	5/22/3	8/18/4	0.83
Follow-up (*x̅* ±s, m)	27.5±9.5	30.2±8.2	0.92
Tumor recurrence (n)			
Intravesical tumor recurrence	3	2	0.32
Extra-bladder recurrence	3	1	0.22

*CTLNU* complete transperitoneal laparoscopic nephroureterectomy, *LNOBE* laparoscopic nephroureterectomy with open bladder cuff excision, *ASA* American Society of Anesthesiologists

To analyze the superiority and feasibility of CTLNU, we evaluated the operative time, blood loss, recovery of bowel function, hospitalization time, and incision length (Table 1). In the CTLNU group, all of these parameters were statistically significantly better than those in the LNOBE group: 30 min less in mean operative time, 90 ml less in mean blood loss, 2 days less in hospital stay length, and 5.2 cm less in mean incision length (all P value < 0.01).

The postoperative pathological examination revealed that the margins of the bladder cuff were negative in all patients, and the T stage of tumors was similar between the two groups. The results showed that this surgical technique could reduce surgical trauma and ensure operative effect simultaneously.

## Discussion

The incidence of UTUC is much lower than that of bladder urothelial carcinoma, accounting for 5 to 7% of all the upper urinary tract tumors [1]. Radical nephroureterectomy is the “golden” surgical procedure to treat UTUC [2, 3]. Nowadays, en bloc dissection of the kidney, ureter, and bladder cuff is the key and standard method for treating UTUC [4, 5].

Laparoscopic nephroureterectomy was first performed by Clayman in 1991, which had been proved as a safe, reproducible, and mini-invasive surgical technique for treating UTUC [6–8]. Because of this technique, postoperative pain, hospital stay, and convalescence period of patients have all decreased [9, 10]. It has been demonstrated that laparoscopic nephroureterectomy (LNU) can achieve the same oncologic efficacy, tumor-free margins, and recurrence rates, regardless of the used laparoscopic approach types [11, 12]. Previous study indicated that the multifocality, T stage 3–4, and G grade 3 were predictors of higher local recurrence rate of UTUC [13]. In our study, the T stage, G grade, location of tumor, and tumor recurrence rate were comparable between the CTLNU and LNOBE groups.

The “standard” LNU technique has not been defined and continues to evolve. To date, a number of methods, including open excision [14], cystoscopic detachment and ligation [15], transurethral resection [16], laparoscopic stapling [17], and ureteric intussusception [18], have been presented for managing the distal ureter in LNU. However, no consensus has been achieved with respect to the best method of approaching the distal ureter and bladder cuff.

There is no need for patients under CTLNU to repositioning, resterilizing, and redraping. Compared with the conventional procedure, our procedure has the following features: (1) the anatomical locations of the trocars are similar to those of the traditional transperitoneal approach and their establishment is relatively easy for surgeons with laparoscopic experience; (2) the transperitoneal approach provides surgeons with the clear anatomical landmarks and wide operating space; (3) with good operating space and vision for bladder incision, surgeons can suture the bladder properly, and bladder catheter can be removed 10 days after surgery; (4) the absence of repositioning and resterilizing saves about 30 min of surgery time; (5) for LNOBE, the median incision of the lower abdomen is needed to expose the lower ureter, while the extended incision for the camera in CTLNU is only used for specimen removal and relatively less invasive; and (6) enlarging the camera port at the lateral margin of rectus abdominis muscle to remove specimens can minimize muscle damage and facilitate postoperative recovery [9, 19].

Compared with LNOBE, there is no need of repositioning, resterilizing, and redraping for CTLNU. The different operation positions can be attended by changing the angels of the operating bed. There are obvious benefits for the patients who undergo the CTLNU: shorter hospital stay, better cosmetic result, and more brief convalescence.

Our study indicated the technique efficiency and initial results were promising. However, the main limitation of our study was its single-institutional retrospective study design. Further studies with more included patients and longer follow-up time are needed to confirm the advantage of this technique.

## Conclusion

Our results suggest that the CTLNU in a single position is technically feasible and safe to manage the distal ureter and bladder cuff. It has advantages of the shorter operation time, less blood loss, and shorter incision length. Our technique makes the complete transperitoneal laparoscopic nephroureterectomy adhering to oncologic principle without need for patients repositioning and offers a complex endoscopic procedure to perform the radical nephroureterectomy.

## Data Availability

The data used and analyzed in this research can be obtained from the corresponding author with a reasonable request.

## Abbreviations

UTUC: Urinary tract urothelial carcinoma
CTLNU: Complete transperitoneal laparoscopic nephroureterectomy
LNOBE: Laparoscopic nephroureterectomy with open bladder cuff excision
LNU: Laparoscopic nephroureterectomy
ASA: American Society of Anesthesiologists

## References

[1] Tan WS, Feber A, Sarpong R, Khetrapal P, Rodney S, Jalil R, Mostafid H, Cresswell J, Hicks J, Rane A (2018). Who should be investigated for haematuria? Results of a contemporary prospective observational study of 3556 patients. Eur Urol..

[2] Li S, Pan Y, Jinghai H (2019). Oncologic outcomes comparison of partial ureterectomy and radical nephroureterectomy for urothelial carcinoma. BMC Urol.

[3] Liu F, Guo W, Zhou X, Ding Y, Ma Y, Hou Y, Kong X, Wang Z (2018). Laparoscopic versus open nephroureterectomy for upper urinary tract urothelial carcinoma: a systematic review and meta-analysis. Medicine (Baltimore).

[4] Seisen T, Peyronnet B, Dominguez-Escrig JL, Bruins HM, Yuan CY, Babjuk M, Böhle A, Burger M, Compérat EM, Cowan NC, Kaasinen E, Palou J, van Rhijn BWG, Sylvester RJ, Zigeuner R, Shariat SF, Rouprêt M (2016). Oncologic outcomes of kidney-sparing surgery versus radical nephroureterectomy for upper tract urothelial carcinoma: a systematic review by the EAU non-muscle invasive bladder cancer guidelines panel. Eur Urol.

[5] Liu W, Wang Y, Zhong Z, Jiang H, Ouyang S, Zhu L, Xu R (2016). Transperitoneal versus retroperitoneal laparoscopic nephroureterectomy in the management of upper urinary tract urothelial carcinoma: a matched-pair comparison based on perioperative outcomes. Surg Endosc..

[6] Clayman RV, Kavoussi LR, Figenshau RS, Chandhoke PS, Albala DM (1991). Laparoscopic nephroureterectomy: initial clinical case report. J Laparoendosc Surg..

[7] Peyronnet B, Seisen T, Dominguez-Escrig J-L, Bruins HM, Yuan CY, Lam T, Maclennan S, N’dow J, Babjuk M, Comperat E, Zigeuner R, Sylvester RJ, Burger M, Mostafid H, van Rhijn BWG, Gontero P, Palou J, Shariat SF, Roupret M (2019). Oncological outcomes of laparoscopic nephroureterectomy versus open radical nephroureterectomy for upper tract urothelial carcinoma: an European Association of Urology guidelines systematic review. Eur Urol Focus.

[8] Zhang C, Wang F, Shi X l, Guo F, Wang H q, Yang Y, Gao X f, Yang B (2018). Direct lateral access to renal artery during transperitoneal laparoscopic partial nephrectomy: surgical technique and comparative outcomes. Urology.

[9] Miyake M, Nishimura N, Aoki K, Ohmori C, Shimizu T, Owari T, Hori S, Morizawa Y, Gotoh D, Nakai Y, Anai S, Torimoto K, Tanaka N, Fujimoto K (2020). Initial experience of complete laparoscopic radical nephroureterectomy combined with transvesical laparoscopic excision of distal ureter in patients with upper urinary tract cancer. World J Surg Oncol.

[10] Chromecki TF, Cha EK, Fajkovic H, Margulis V, Novara G, Scherr DS, Lotan Y, Raman JD, Kassouf W, Bensalah K, AlonWeizer EK, Roscigno M, Remzi M, Matsumoto K, Walton TJ, Pycha A, Ficarra V, Karakiewicz PI, Zigeuner R, Pummer K, Shariat SF (2012). The impact of tumor multifocality on outcomes in patients treated with radical nephroureterectomy. EurUrol.

[11] Xylinas E, Rink M, Cha EK, Clozel T, Lee RK, Fajkovic H, Comploj E, Novara G, Margulis V, Raman JD, Lotan Y, Kassouf W, Fritsche H-M, Weizer A, Martinez-Salamanca JI, Matsumoto K, Zigeuner R, Pycha A, Shariat SF (2014). Impact of distal ureter management on oncologic outcomes following radical nephroureterectomy for upper tract urothelial carcinoma. Eur Urol..

[12] Piszczek R, Nowak Ł, Krajewski W, Chorbińska J, Poletajew S, Moschini M, Kaliszewski K, Zdrojowy R (2021). Oncological outcomes of laparoscopic versus open nephroureterectomy for the treatment of upper tract urothelial carcinoma: an updated meta-analysis. World J Surg Oncol.

[13] Li X, Cui M, Xiaobin G, Dong F, Li H, Qin S, Yang K, Zhu T, Li X, Zhou L, Gao X-S, Wang D (2020). Pattern and risk factors of local recurrence after nephroureterectomy for upper tract urothelial carcinoma. World J Surg Oncol.

[14] Tsivian A, Benjamin S, Sidi AA (2007). A sealed laparoscopic nephroureterectomy: a new technique. European Urology.

[15] Gill IS, Sung GT, Hobart MG, Savage SJ, Meraney AM, Schweizer DK, Klein EA, Novick AC (2000). Laparoscopic radical nephroureterectomy for upper tract transitional cell carcinoma: the Cleveland Clinic experience. J Urol.

[16] Ubrig B, Boenig M, Waldner M, Roth S (2004). Transurethral approach to the distal ureter in nephroureterectomy: transurethral extraction vs. “pluck” technique with long-term follow-up. Eur Urol.

[17] Tan BJ, Ost MC, Lee BR (2005). Laparoscopic nephroureterectomy with bladder-cuff resection: techniques and outcomes. J Endourol.

[18] Laguna MP, de la Rosette JJ (2001). The endoscopic approach to the distal ureter in nephroureterectomy for upper urinary tract tumor. J Urol.

[19] Zhang X, Wang K, Ma J, Zhang Q, Liu C, Cui Y, Lin C (2019). Total laparoscopic nephroureterectomy for upper urinary tract urothelial carcinoma under a single surgical position. World J Surg Oncol.

